# Beyond the Tooth‐Bearing Zone: Unusual Presentation of an Odontogenic Fibromyxoma in the Mandibular Condyle

**DOI:** 10.1155/crot/1700218

**Published:** 2026-05-25

**Authors:** Karl El Mendelek, Amjad Damaj, Jihad El Khoury

**Affiliations:** ^1^ Department of Otolaryngology - Head and Neck Surgery, Saint George Hospital University Medical Center, Beirut, Lebanon, stgeorgehospital.org

**Keywords:** alveolar process, mandibular condyle, odontogenic fibromyxoma, tooth-forming apparatus

## Abstract

Odontogenic fibromyxoma is a rare benign tumor that arises from mesenchymal components of the tooth‐forming apparatus and typically occurs in the alveolar process of the jaws. We are reporting an atypical case of an incidental tumor located in the mandibular condyle of a child, an area not associated with odontogenic tissue. The lesion was identified through imaging and confirmed histopathologically after surgical excision. To our knowledge, this is the first reported case of an odontogenic fibromyxoma in the condyle of a pediatric patient.

## 1. Introduction

A myxoma is a benign tumor composed of a gelatinous, mucus‐like substance due to its abundant mucoid stroma, as defined by the World Health Organization (WHO) [1, 2]. It arises from undifferentiated mesenchymal cells that undergo myxoid differentiation, producing glycosaminoglycans instead of typical connective tissue fibers, resulting in a loose, mucinous matrix [3]. Fibromyxoma is a subtype characterized by increased fibrous content, although the WHO uses both terms interchangeably, making epidemiological distinction difficult [1, 4]. Odontogenic fibromyxoma refers to a tumor that originates specifically from the odontogenic ectomesenchyme, derived from neural crest cells. The latter gives rise to the dental papilla and dental follicle, which constitute the major precursors for tooth development. In the WHO classification of head and neck tumors, last revised in 2022, odontogenic fibromyxoma can be categorized as central (intraosseous), exhibiting locally aggressive features, or peripheral (extraosseous) with less aggressive behavior. This rare tumor accounts for less than 4% of myxomas and less than 8% of all odontogenic neoplasms of the jaw [1, 2, 5]. While all age groups can be affected, this neoplasm is most commonly encountered between the second and fourth decades of life [2, 5–8]. Odontogenic fibromyxoma, as its name implies, develops on tooth‐bearing areas of the jaw, with a mild tendency for it to localize in the posterior mandible [8].

## 2. Case Report

We are highlighting the case of a 9‐year‐old boy, with no known food or drug allergies, and no previous medical or surgical concerns, who presented for checkup after an uncomplicated head trauma; he had no complaints and no symptoms. History went back to 1 week prior to his presentation when he was subject to an accidental head trauma as he fell down the stairs. His parents described the trauma as mild to moderate in intensity as it was uneventful and denies any resultant bruises or lacerations or abnormal behavioral or neurological symptoms back then. However, for further reassurance, the child was taken to the emergency department at another institution where he underwent a computed tomography (CT) scan of the brain which came out negative for any acute intracranial events but described an incidental expansile mass in the left mandibular condyle associated with cortical erosion, more so medially, measuring 2 cm in height, 1.6 cm anteroposteriorly, and 1.5 cm transversally (Figure 1).

**FIGURE 1:**
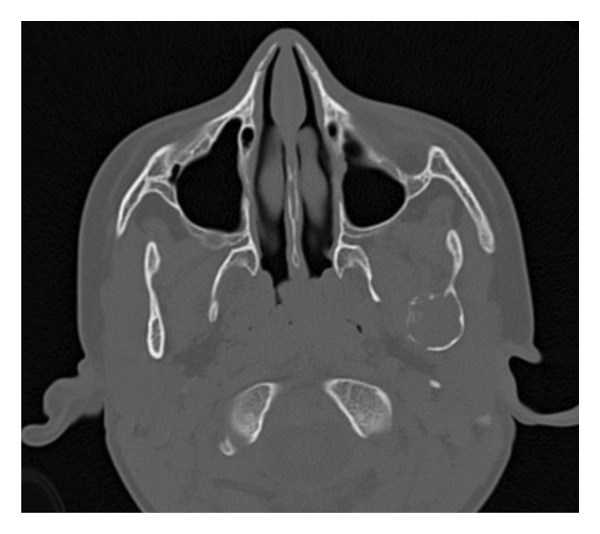
Brain CT showing left mandibular condyle lesion with medial cortical erosion.

In front of this incidental finding, the patient was brought for further evaluation. On physical exam and inspection, the child exhibited symmetrical facial features and expressions, a normal mouth opening of around 45 mm measured using a manual caliper, normal dental occlusion, and no evidence of bulging, tenderness, or erythema overlying the left temporomandibular joint (TMJ). Oral and oropharyngeal examination was also nonsignificant, as he had normal appearance of the buccal mucosa and unremarkable dental condition. Therefore, the evaluation was completed by a panoramic radiograph which showed a unilocular, well‐demarcated radiolucency, extending from the left mandibular condyle to the midportion of the ramus. Magnetic resonance imaging (MRI) of the brain was also requested for better characterization. It showed the lesion demonstrating intermediate signal intensity on T1‐weighted sequence, intermediate to high signal intensity on T2‐weighted sequence, and diffuse enhancement after the administration of intravenous gadolinium (IV G) injection. Figures 2 and 3 illustrate the aforementioned imaging studies.

**FIGURE 2:**
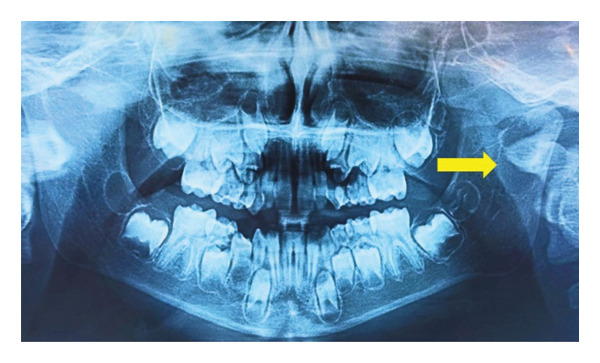
Panoramic radiograph showing unilocular lesion in left mandibular condyle (arrow).

**FIGURE 3:**
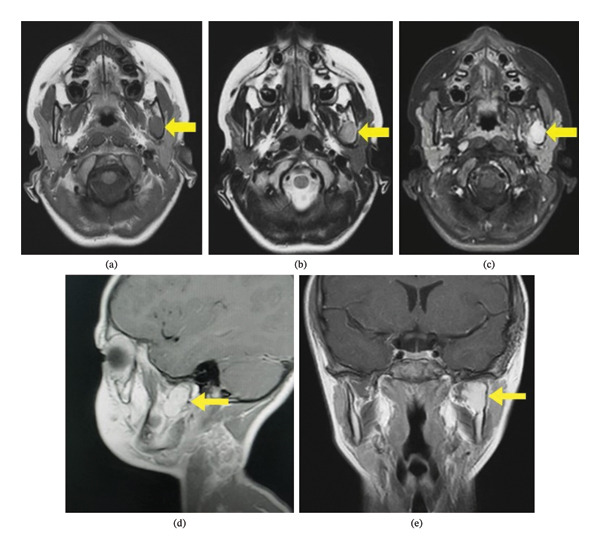
Brain MRI showing left condylar lesion (yellow arrow): (a) axial cut, T1‐weighted; (b) axial cut, T2‐weighted; (c) axial cut, T1‐weighted + IV G; (d) sagittal cut, T1‐weighted + IV G; (e) coronal cut, T1‐weighted + IV G.

Imaging was nondefinitive; thus, surgical intervention was planned after discussing with the parents, for both diagnostic and therapeutic purposes. The patient was taken to the operating room where surgery was conducted under general anesthesia. Through a preauricular approach, dissection was carried carefully, preserving the facial nerve, until the left TMJ was reached. The articular disc was identified and inspected; it appeared macroscopically intact, with no evidence of distortion, invasion, or adherence to the lesion. The lesion was completely confined within the bony condyle, allowing safe dissection and preservation of the articular disc. The lesion was dissected from the center of the condyle and was sent for frozen section analysis which turned out inconclusive, but confirmed the benign aspect of the lesion. After performing curettage of the resultant bony cavity and drilling of the bony margins using a diamond burr, the surgery was concluded. Postoperatively, the patient had an uneventful clinical course, with no facial paralysis and normal mouth opening. He was prescribed prophylactic amoxicillin–clavulanate to prevent wound infection for 1 week, systemic prednisone with a taper to decrease edema for 1 week, and analgesics as needed. He was also educated to have a soft diet for 1 month to prevent fracture of the hollow condyle by the time new ossification occurs. Pathology results came back 10 days after surgery. Figure 4 shows the histopathological slide. The specimen was stained with hematoxylin and eosin (H&E). Histopathological examination revealed a hypocellular neoplasm composed mainly of spindle‐shaped monotonous bland cells embedded in an abundant myxoid extracellular matrix, alternating with more cellular areas of fibroblasts with collagenous stroma, consistent with a fibromyxoid pattern. The lesion demonstrated infiltrative growth into adjacent bony trabecula. No cytologic atypia, necrosis, or increased mitotic activity was observed. Surgical margins were not assessable due to curettage. Immunohistochemistry came back as follows: vimentin (positive), cytokeratins (negative), S‐100 (negative), CD34 (negative), desmin/SMA (negative), and Ki‐67 (low proliferation). The histological differential diagnosis included odontogenic fibroma, central myxoma, neurofibroma, and myxoid sarcoma. No odontogenic epithelial remnants were identified. However, their absence does not preclude the diagnosis, as odontogenic fibromyxomas frequently lack epithelial components. The diagnosis was made based on characteristic histomorphological features and exclusion of other myxoid neoplasms.

**FIGURE 4:**
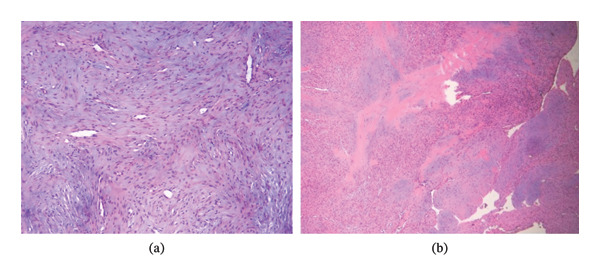
Histopathological slide stained with H&E: (a) High‐power view showing spindle‐shaped cells embedded in abundant myxoid stroma with focal fibrous differentiation; (b) low‐power view demonstrating infiltrative growth between residual bony trabecula.

The patient was reassessed in the office at 1 month, 6 months, and 1 year after surgery. Postoperative follow‐up was satisfactory in all visits, with normal mouth opening and dental occlusion, preserved facial nerve function, and normally healing wound. For radiological assessment, the first panoramic radiograph was done at 6 months, as shown in Figure 5. It demonstrated the left mandibular condyle with restored continuity and preserved overall morphology. The previously described radiolucent expansile lesion was no longer visualized, but evidence of progressive bone remodeling was noted, characterized by partial reossification of the prior curettage cavity, increased radiodensity within the condylar head compared to the preoperative imaging, and smooth cortical outline, with no signs of cortical breach or irregularities. In view of the patient’s young age, a structured follow‐up protocol was recommended, consisting of panoramic radiographs every 6 months for the first 2 years and annually thereafter for a minimum of 5 years. Any concerning clinical or radiologic findings were to be further investigated with a more advanced imaging modality.

**FIGURE 5:**
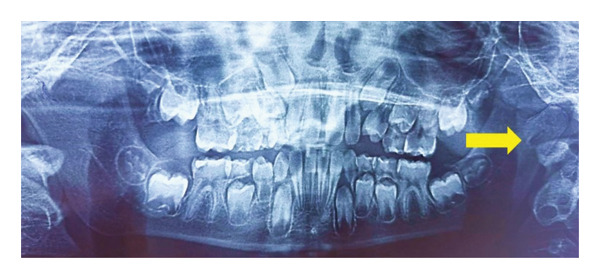
Panoramic radiograph at 6 months postoperatively showing the left mandibular condyle with preserved morphology and evidence of bone remodeling (arrow).

## 3. Discussion

Histologically, fibromyxomas can present in soft tissue or bones, with the heart being the most commonly involved organ overall. Odontogenic fibromyxoma is a rare, benign intraosseous tumor primarily affecting the tooth‐bearing regions of the maxilla and mandible, with a predilection for the molar and premolar areas of the mandible. Though nonmetastasizing, it can be locally aggressive with destructive behavior [5, 9], as shown in our patient in whom the lesion demonstrated beginning of medial cortical erosion. The tumor typically peaks in individuals in their thirties, with female predominance reported across multiple studies. However, occasional cases in males, and in the pediatric age particularly, such as the present one involving a 9‐year‐old boy, challenge this trend. A review of pediatric cases under 10 years of age revealed just a small number of odontogenic fibromyxomas in the mandible [5]. Some authors, like Zayet et al., suggest that the tumor may originate from mesenchymal rests within the alveolar processes of the jaws [10]. Other regions of the mandible, like the ramus or condyle, have not been implicated as potential sites for odontogenic tumors.

Clinically, odontogenic fibromyxoma typically presents as a slow‐growing, insidious, and oligosymptomatic lesion [11, 12]. This is concordant with our case whose lesion was completely incidental and asymptomatic. Despite its subtle onset, it sometimes leads to thinning or perforation of the surrounding cortical bone, causing swelling, tooth displacement, malocclusion, facial symmetry, or paresthesia. Radiographically, the tumor can display a wide range of appearances, often mimicking other jaw lesions and render accurate diagnosis challenging. Usually, it appears well‐demarcated and radiolucent, unilocular in case of small lesions, multilocular with internal septations in case of larger ones, giving a classic “honeycomb” or “tennis racket” appearance [13, 14]. Remarkably, the tumor of our patient, despite its relatively large size, lacked internal septations. Given their radiological variability, odontogenic fibromyxomas are often misdiagnosed and must be differentiated from lesions like ameloblastomas, central giant cell granulomas, intraosseous hemangiomas, and myxoid sarcomas, which present radiological and clinical similarities [14]. Therefore, histopathological confirmation remains essential for a definitive diagnosis.

What makes our case especially notable is the location: the mandibular condyle, a non–tooth‐bearing area. To date, reports of odontogenic fibromyxoma in non–tooth‐bearing regions such as the ramus–condyle unit are exceedingly rare. In fact, published literature identifies only a few cases involving this area [15]. The condyle lacks the odontogenic ectomesenchyme which is usually implicated in the origin of odontogenic fibromyxomas. This raises questions about histogenesis in such an atypical location and whether an odontogenic tumor can truly develop in areas devoid of classic odontogenic tissue. Several theories may account for this unusual presentation. One possibility is the presence of ectopic odontogenic rests that may have been trapped during embryogenesis, although this remains speculative. Alternatively, it is conceivable that the lesion represents an odontogenic fibromyxoma from the adjacent ramus with secondary condylar involvement, although our surgical findings in this patient do not support this theory. Another possibility is misdiagnosis; however, the definitive histopathologic features and immunohistochemical studies of our case confirmed the diagnosis.

Management of an odontogenic fibromyxoma in the condylar region, particularly in a growing child, presents clinical challenges. Given the tumor’s locally aggressive behavior and tendency to recur, complete surgical excision with clear margins is generally recommended [12, 14]. However, in selected patients, when the tumor involves the mandibular condyle, there is critical need to balance between tumor control and preservation of jaw growth and function [9, 16]. This is particularly the case in pediatric patients. Although the lesion in our patient was asymptomatic, a conservative observational strategy was not appropriate due to the radiologic findings of cortical erosion and expansile behavior, suggesting a locally aggressive tumor. Furthermore, imaging alone was insufficient to establish a definitive diagnosis, and histopathological analysis was necessary to exclude other potentially aggressive or malignant lesions. In pediatric patients, delayed management of such lesions may result in further bony destruction, potential impairment of mandibular growth, and increased surgical complexity. Therefore, early surgical intervention was deemed justified despite the known risks associated with condylar surgery in a child. Marginal or segmental mandibulectomy at the level of the condyle may have long‐term implications for TMJ function and occlusion. In our case, a conservative surgical approach was favored, with close postoperative monitoring to detect any early signs of recurrence.

The rarity of this tumor in the condylar region also highlights the broader issue of tumor classification in the craniofacial skeleton. While the WHO classifies it as an odontogenic tumor, our present case suggests that our understanding of the potential sites of origin may need to be reconsidered. Further documentation of similar cases, if encountered, will be essential to refine diagnostic criteria and treatment protocols.

## 4. Conclusion

In conclusion, this case represents what appears to be the first reported instance of odontogenic fibromyxoma in the mandibular condyle of a child. Its discovery in a non–tooth‐bearing region challenges the conventional understanding of the tumor’s origin and behavior. Clinicians should be aware of the possibility of odontogenic tumors presenting in atypical locations, particularly when evaluating radiolucent lesions in pediatric patients. This case expands the known clinical spectrum of odontogenic fibromyxomas and serves as a reminder of the diagnostic surprises that can occur even in well‐characterized tumor types.

## Author Contributions

Karl El Mendelek, M.D, wrote the manuscript, collected data, and performed literature review. Amjad Damaj, M.D, helped in literature review. Jihad El Khoury, M.D, the senior author, supervised the work.

## Funding

This research received no external funding.

## Consent

The patient’s parents provided a written informed consent for publication of the case.

## Conflicts of Interest

The authors declare no conflicts of interest.

## Data Availability

The data that support the findings of this study are available from the corresponding author upon reasonable request.
